# Supplementary material for: Structural basis for DNA 3′-end processing by human tyrosyl-DNA phosphodiesterase 1
Source: Nat Commun. 2018 Jan 2;9:24. doi: 10.1038/s41467-017-02530-z (PMC5750209; doi:10.1038/s41467-017-02530-z)
Supplement: Supplementary file 1 — Supplementary Figures [file 41467_2017_2530_MOESM1_ESM.pdf]

Structural basis for DNA 3'-end processing by human Tyrosyl-DNA phosphodiesterase 1

Supplementary Figures

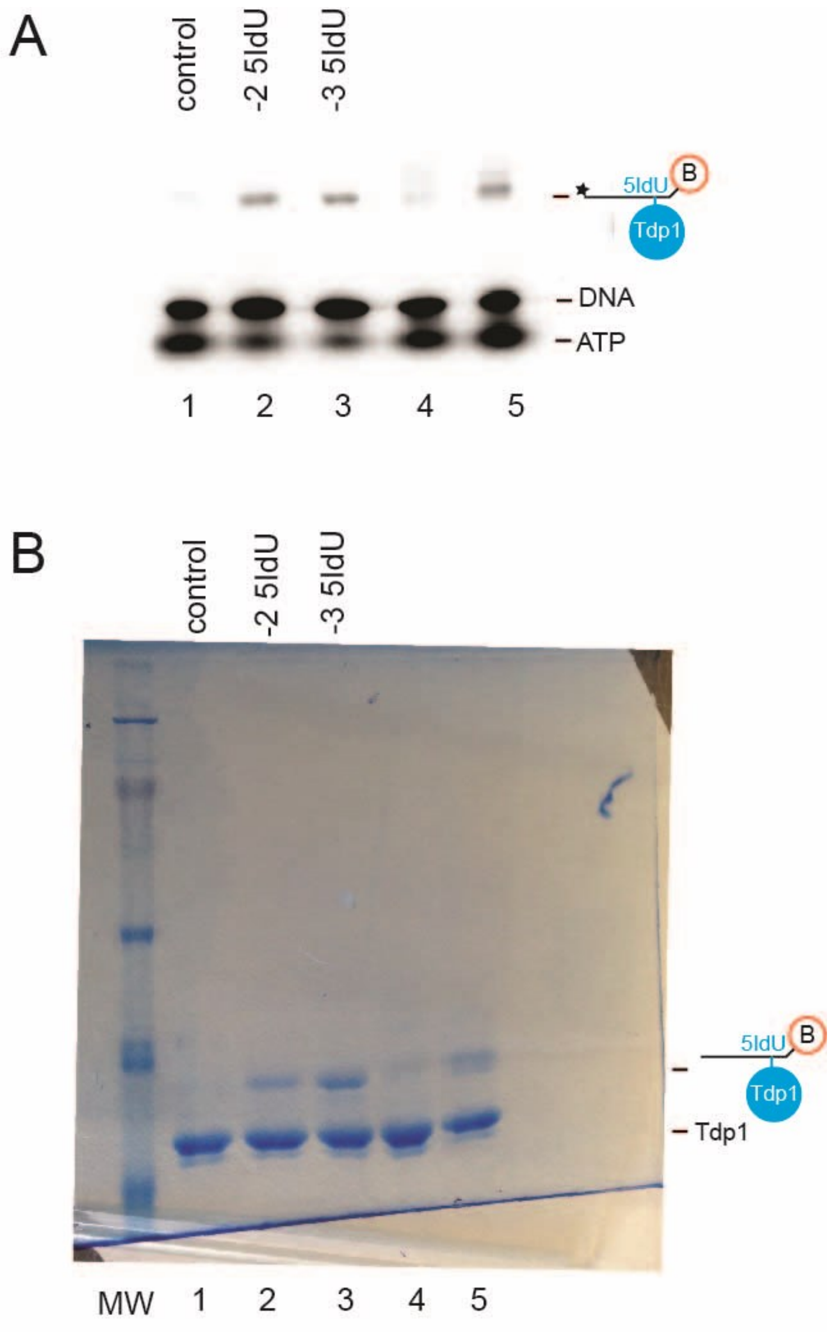

Supplementary Figure 1. Uncropped images used to produce Figure 3B. 8% SDS PAGE of DNA oligonucleotides, containing a ^{32}P-label, cross-linked to catalytically inactive Tdp1(Δ148) H263A. (A) Visualised by phosphorimaging (upper panel in Figure 3B) and (B) by SimplyBlue™ staining (lower panel in Figure 3B). Lanes 1-3 contain reactions with unmodified control oligonucleotide, -2 5IdU and -3 5IdU oligonucleotides, respectively. Lanes 4 and 5 contain samples not related to this project.

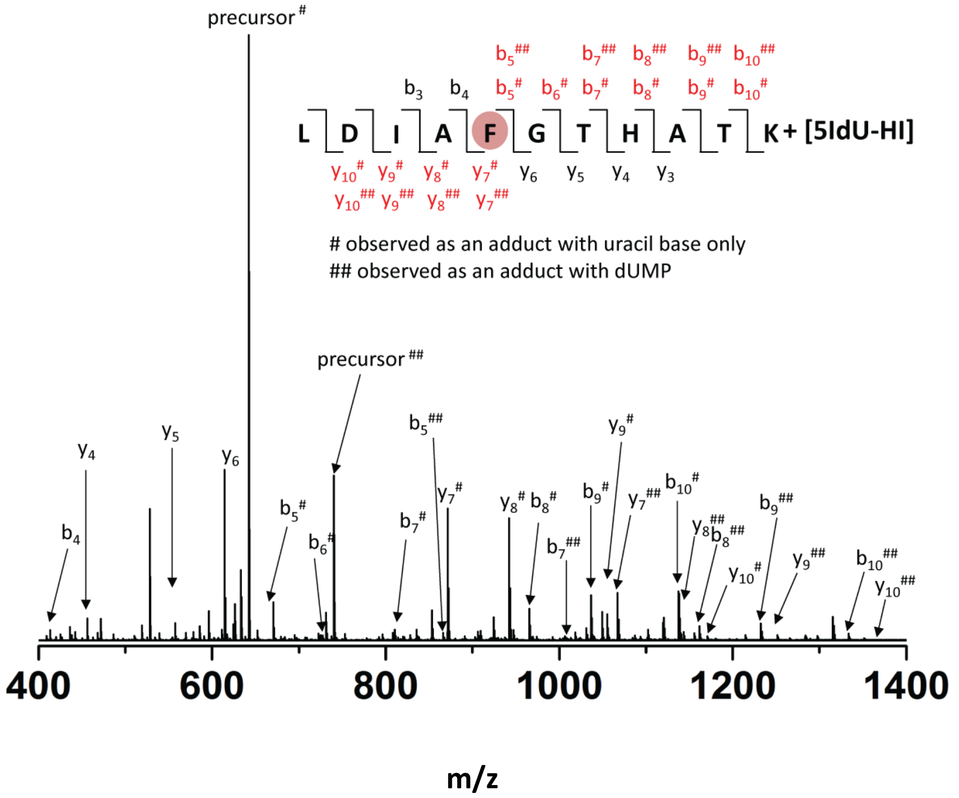

Supplementary Figure 2. Tdp1(Δ148) cross-links *via* F259 to the -3 modified nucleobase. Collision induced dissociation fragmentation (CID) mass spectrum of the $[M+2H]^{2+}$ ion at *m/z* 740.33 at 20 V (for the -3 cross-link sample) and the sequence of the cross-linked Tdp1 peptide. The position of the DNA cross-link (F, shaded in red) is determined by the presence of modified fragment ions (annotated in red) containing a single deoxyuracil monophosphate (dUMP, annotated with ##) or a uracil base (annotated by #), which arise from fragmentation of the glycosidic ribose–base bond during CID. Identified peptide *b* and *y* fragment ions are annotated on the spectrum in black.

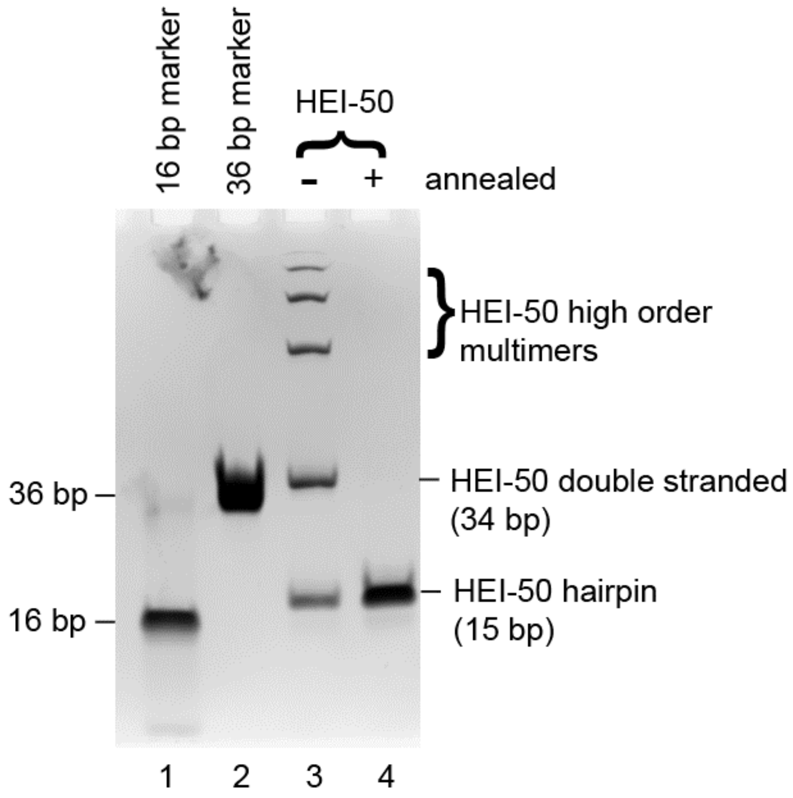

Supplementary Figure 3. Native polyacrylamide gel of the HEI-50 DNA substrate used in the fluorescence-based Tdp1(Δ148) cleavage assays. 12% (w/v) native polyacrylamide gel lane 1: 16 bp control oligonucleotide, lane 2: 36 bp control oligonucleotide. The 34 nt HEI-50 oligonucleotide before (lane 3) and after (lane 4) rapid annealing.

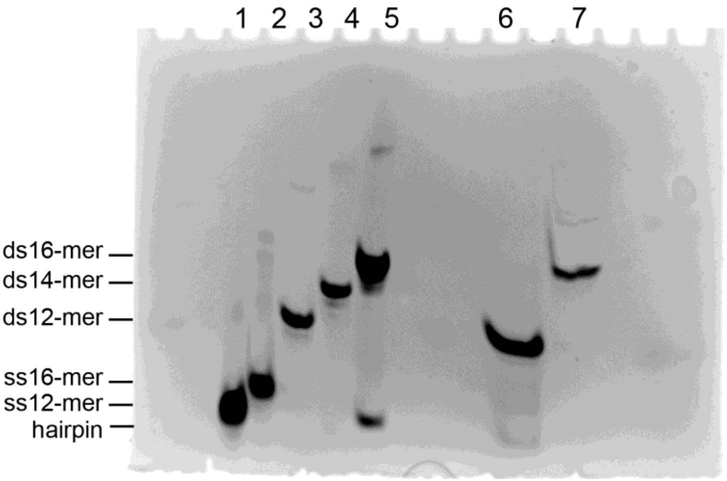

Supplementary Figure 4. **Uncropped image used to produce Figure 5A.** 20 % (w/v) native polyacrylamide gel of DNA oligonucleotides stained with methylene blue. Lanes 1 and 2: single-stranded (ss) 12-mer and 16-mer, respectively; lanes 3, 4 and 5, double-stranded (ds), self-complementary 12-mer, 14-mer and 16-mer, respectively. Lanes 6 and 7 contain DNA samples not related to this project.

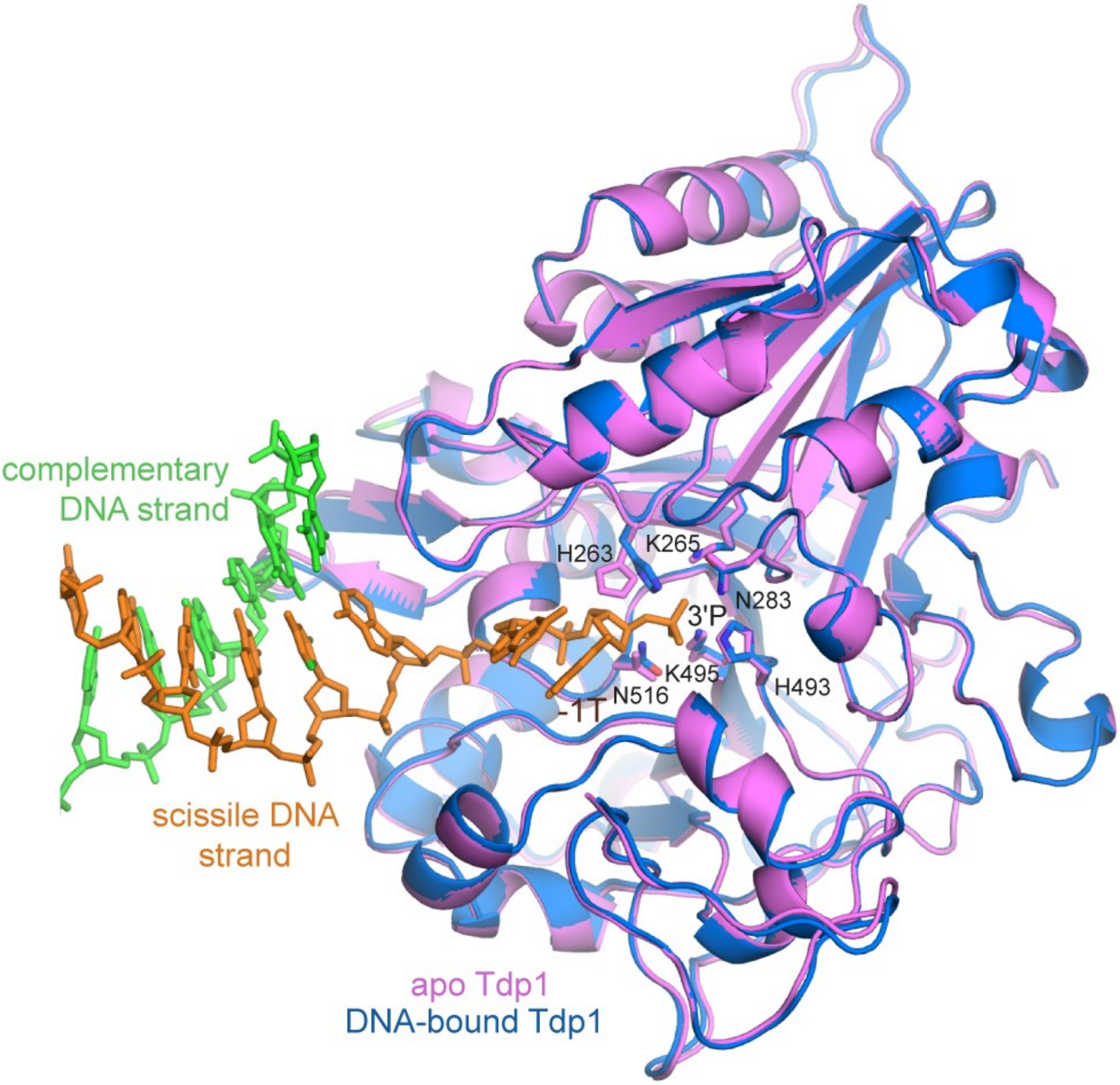

Supplementary Figure 5. Superposition of the two Tdp1(Δ148) molecules in the asymmetric unit of the -2T Tdp1(Δ148):DNA structure. The DNA-bound Tdp1(Δ148) (chain A) is coloured blue, whereas the apo (DNA-free) Tdp1(Δ148) (chain B) is coloured violet. The scissile (orange) and complementary (green) DNA strands are labelled. The active site residues are shown as sticks. Chains A and B superpose with a r.m.s.d. of 0.33 Å over 432 Cα atoms.

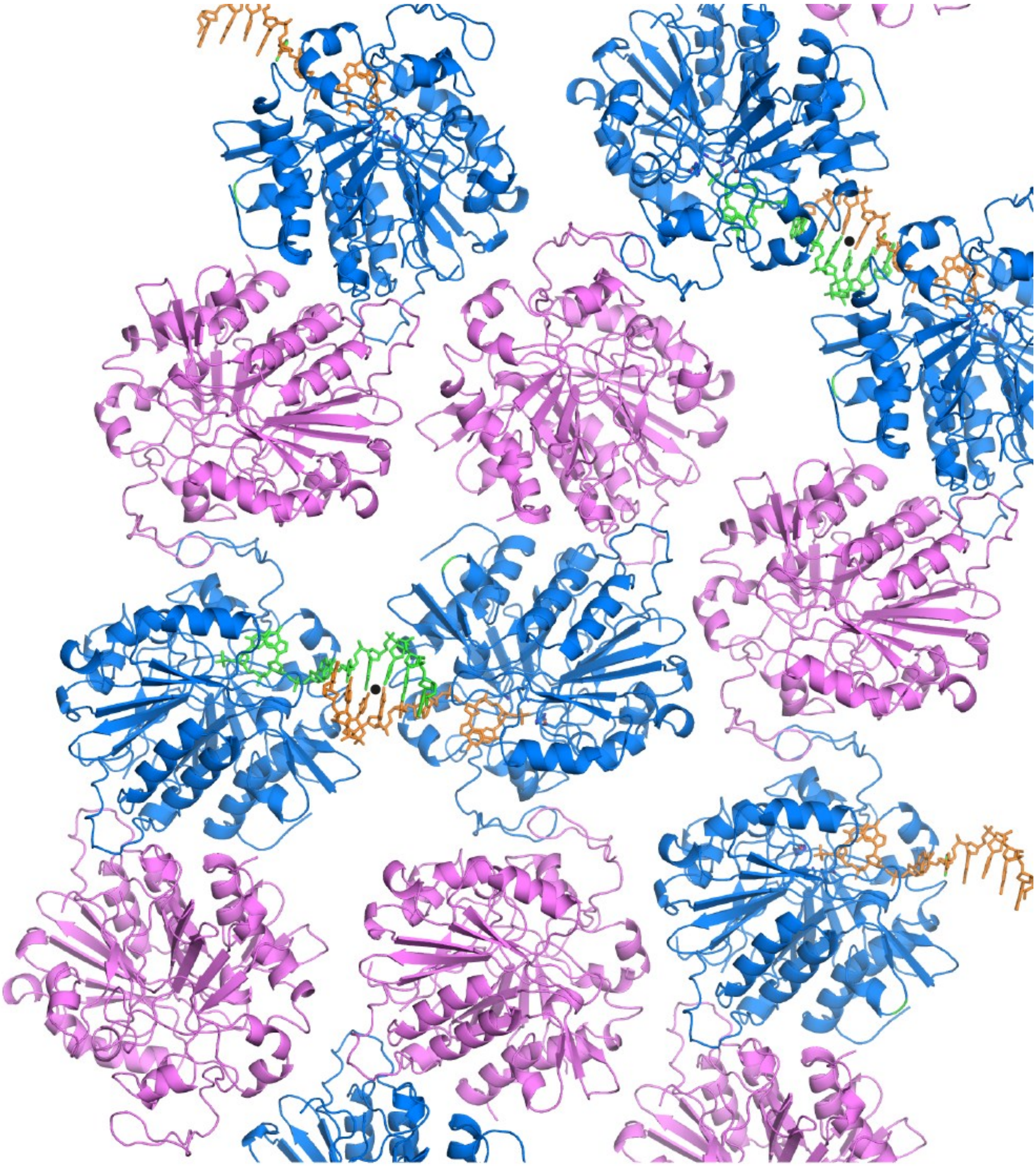

Supplementary Figure 6. Crystal packing arrangement and DNA interactions in the crystal lattice. Each duplex DNA (orange and green) connects two Tdp1(Δ148) molecules (blue) in adjacent asymmetric units. The dyad axis of the symmetrical DNA duplex is coincident with the crystallographic 2-fold symmetry axis (marked by a black dot). Each asymmetric unit also contains a second apo Tdp1(Δ148) molecule (violet).

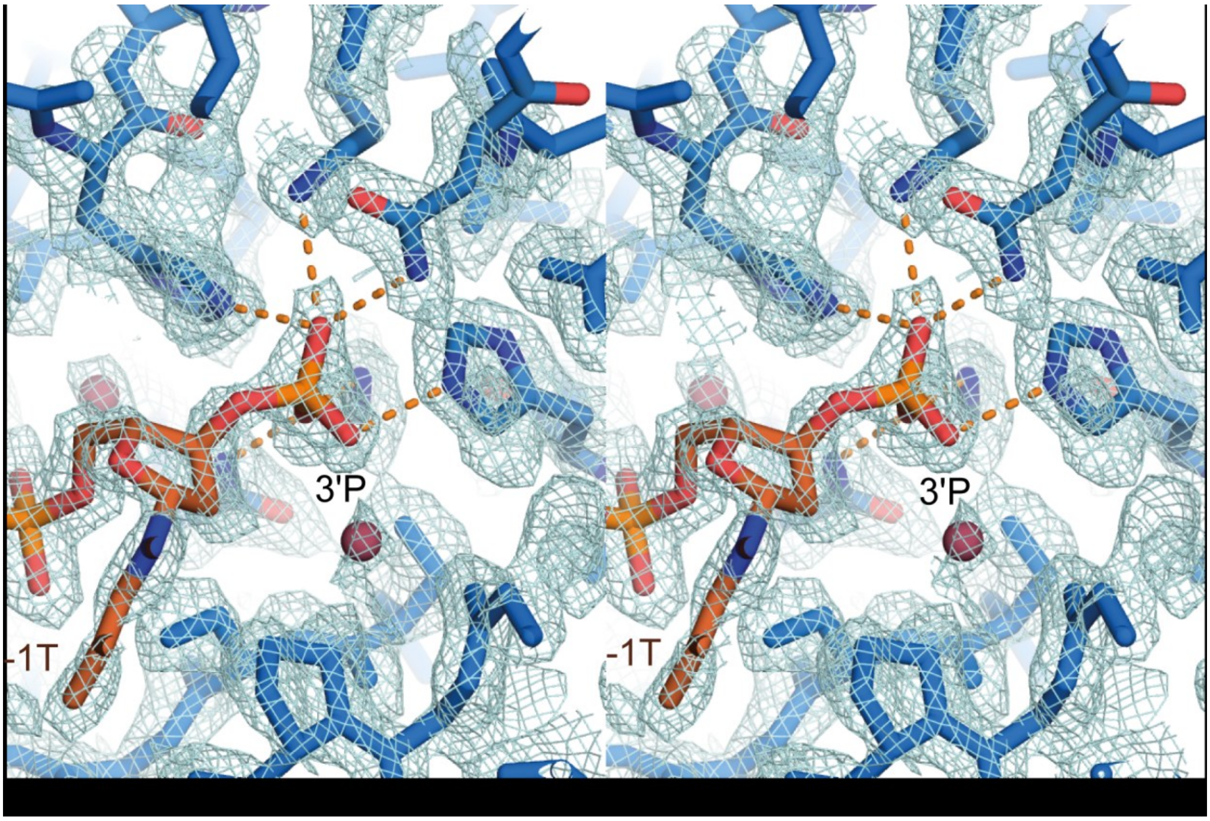

Supplementary Figure 7. Stereo view of the image shown in Figure 5D of the Tdp1(Δ148) active site. Hydrogen bonds to the 3'-phosphorylated thymine (-1T) are shown as orange dashed lines and the water molecule as a red sphere. The $2F_o$-F_c electron density map is shown as a blue mesh contoured at 1.6σ.

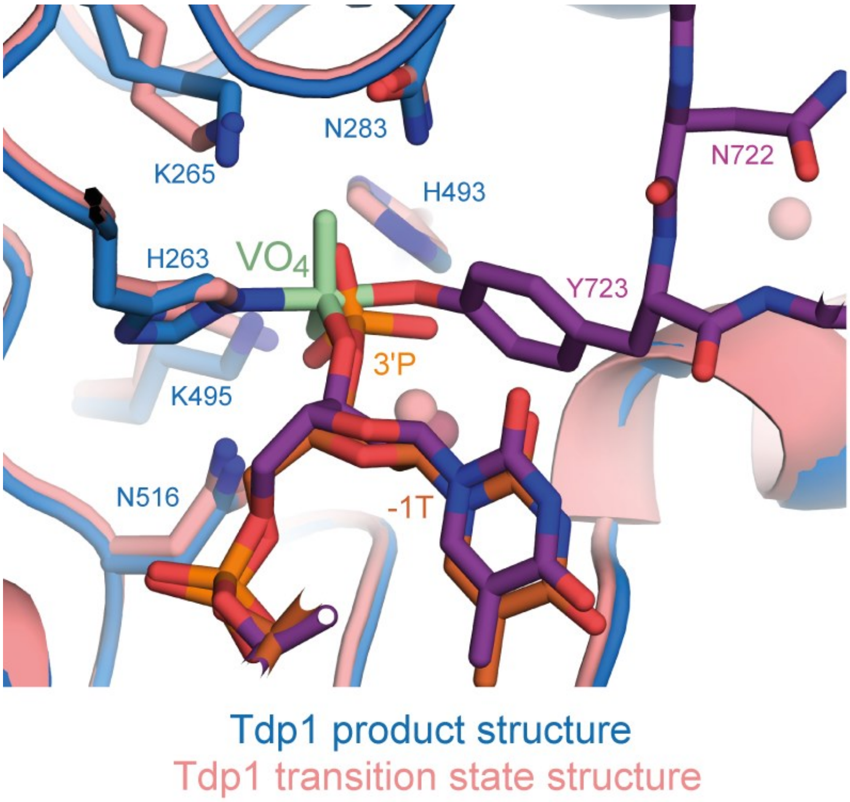

Supplementary Figure 8. Superposition of the Tdp1(Δ148) product and transition-state mimic structures. The product structure, containing Tdp1(Δ148) (blue) bound to -2T duplex DNA (orange) (PDB ID: 5NWA), superposed on the transition-state mimic structure (PDB ID: 1NOP): a quaternary complex containing Tdp1(Δ148) (pink) bound to 3nt of single-stranded DNA (purple), vanadate (V, light grey) and a Top1 peptide fragment (purple). The active site amino acids are labelled. The DNA-bound protein chains in the two structures superpose with an r.m.s.d. of 0.28 Å over 422 Cα atoms.

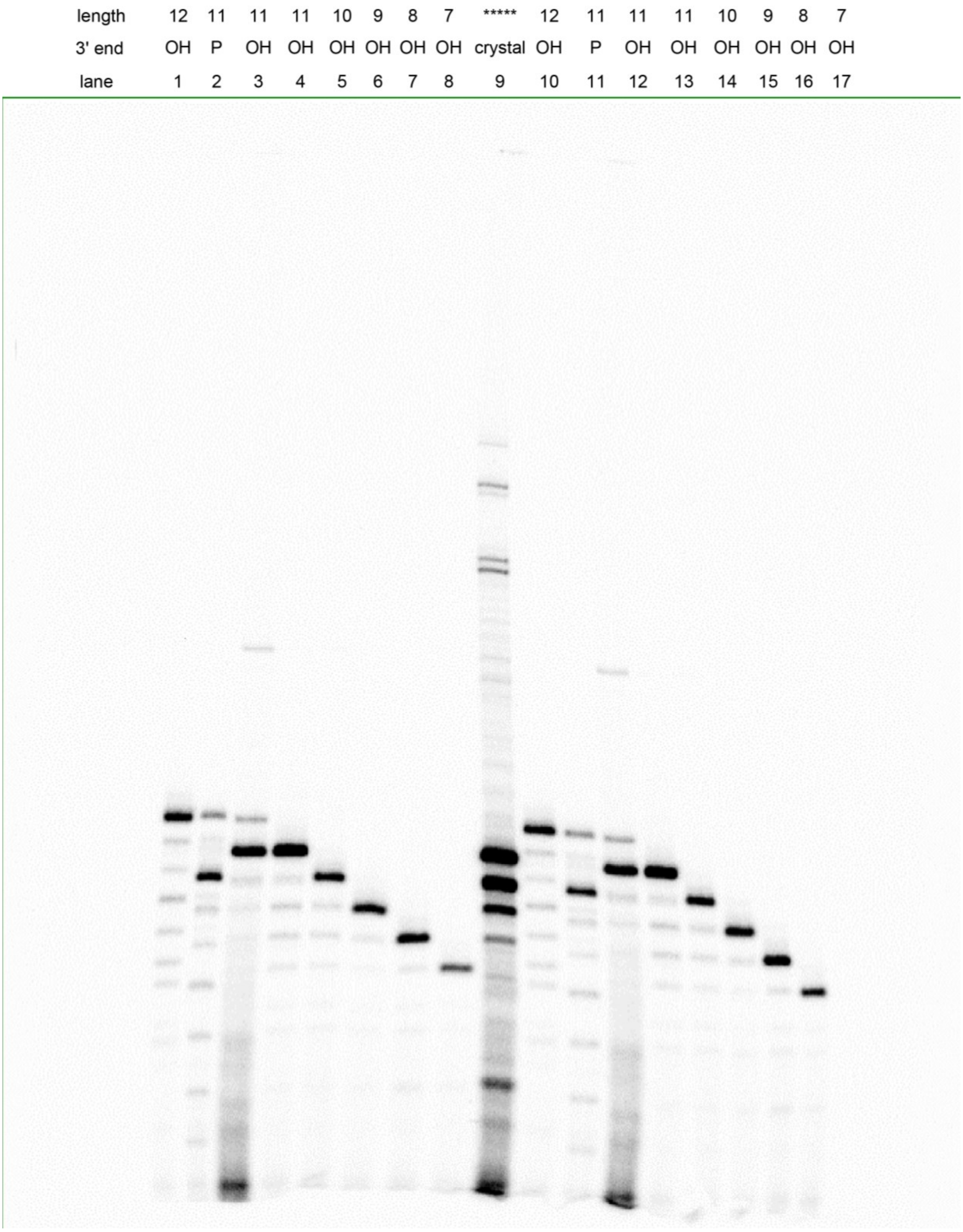

Supplementary Figure 9. Uncropped image of the gel used to produce Figure 6B. Denaturing PAGE of the substrate (lane 1) and products of the reactions shown in Figure 6A (lane 2) and B (lane 3). Lanes 4 to 8 contain a ^{32}P-labelled marker of length 11 nts to 7 nts, respectively. Lane 9 contains the PNK-treated dissolved crystals. Lanes 10 to 17 are repeats of the reactions and markers shown in

lanes 1 to 8.

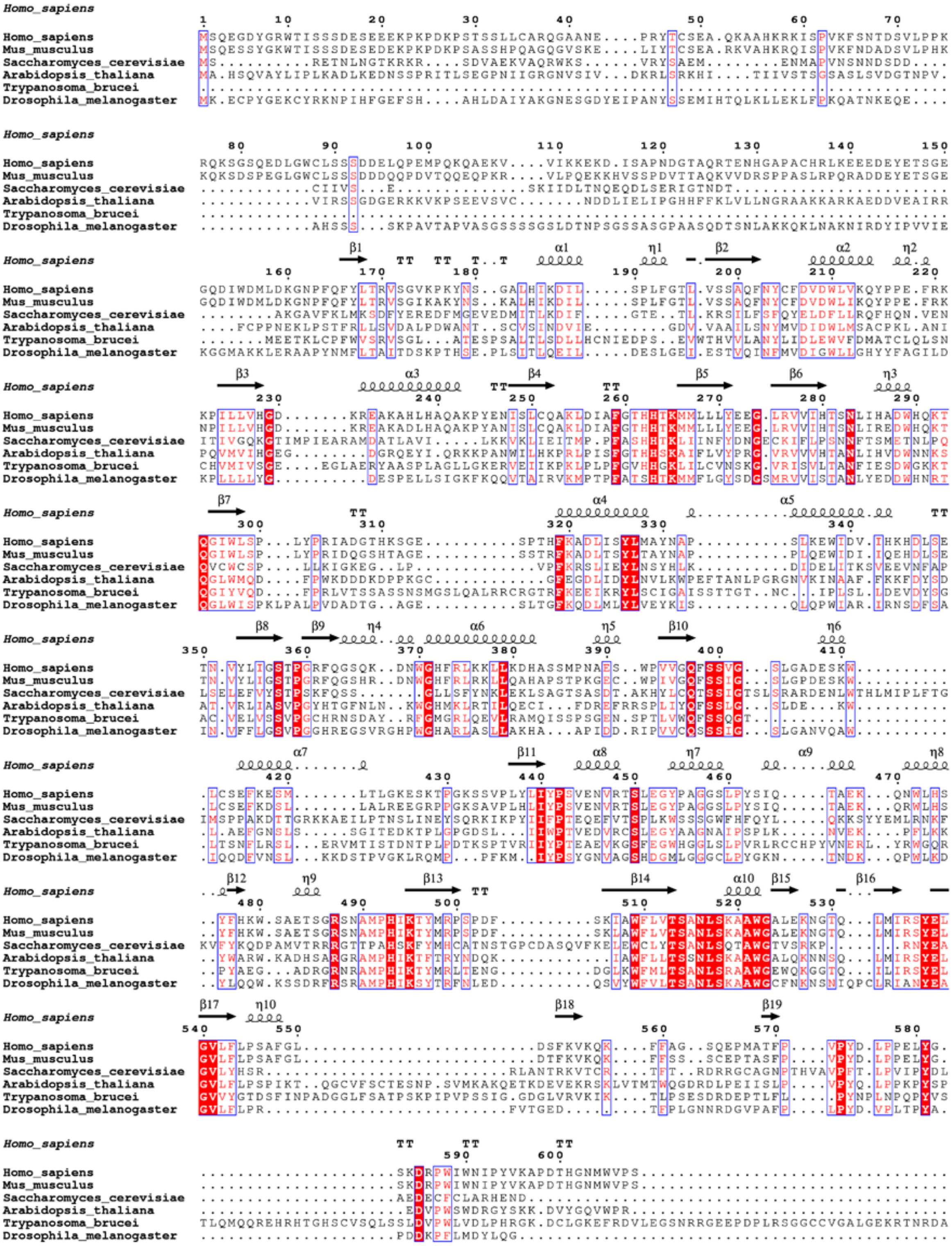

Supplementary Figure 10. Alignment of Tdp1 amino acid sequences. The secondary structure elements of human Tdp1(Δ148) in complex with DNA duplex -2G is shown above the alignment. Sequences are from *Homo sapiens* (NCBI reference sequence: NP_001008744.1), *Mus musculus* (NCBI reference sequence: NP_082630.2), *Saccharomyces cerevisiae* (GenBank: KZV13308.1),

Arabidopsis thaliana (NCBI reference sequence: NP_197021.2)*, Trypanosoma brucei* (GenBank: AAX78999.1) and *Drosophila melanogaster* (NCBI reference sequence: NP_523465.2). Strictly conserved residues are indicated by white lettering on a red background, and partially conserved residues are in red lettering. Catalytic residues are highlighted with a blue circle, K527 with a blue star, and F259 with a purple square. The figure was created with ESPript 3.0 (http://espript.ibcp.fr/ESPript/cgi-bin/ESPript.cgi).
